# Isolated Agenesis of the Splenium of the Corpus Callosum Associated With Autism Spectrum Disorder: A Rare and Clinically Relevant Association

**DOI:** 10.7759/cureus.108487

**Published:** 2026-05-08

**Authors:** Claudio Roberto Ibanez Escalante, Huseyin Dogu Goker, Peter G Bernad

**Affiliations:** 1 Neurology, Universidad Católica de Santa María Facultad de Medicina Humana, Arequipa, PER; 2 Neurology, Sociedad de Investigación en Neurociencias Aplicadas y Psiquiatría (SINAPSIS) Research, Arequipa, PER; 3 Orthopedics and Traumatology, Istanbul University School of Medicine, Istanbul, TUR; 4 Neurology, Neurology Services, Inc, Washington, DC, USA

**Keywords:** agenesis of corpus callosum, autism spectrum disorder, brain connectivity, neurodevelopmental disorders, sleep apnea, splenium

## Abstract

The corpus callosum (CC) is the largest structure of white matter, connecting the two cerebral hemispheres. Agenesis of the corpus callosum (AgCC) is a rare but well-recognized neurodevelopmental malformation. It can be caused by genetic and chromosomal anomalies, metabolic and toxic causes, intrauterine developmental disorders, hypoxic-ischemic insults, infections, and other causes. While attention deficit hyperactivity disorder (ADHD) is frequently associated with AgCC, there is also a significant and disproportionate prevalence of autism spectrum disorder (ASD) in these individuals compared to the general population. We report a rare case with isolated agenesis of the splenium of the corpus callosum associated with autism spectrum disorder in a high-functioning 21-year-old man, who was brought to the neurology clinic by his stepmother with severe sleep dysregulation, excessive daytime sleepiness, and progressively aggressive behavioral issues. It is noteworthy that his biological mother was diagnosed with ADHD and has a cocaine addiction. Laboratory findings were within normal limits, and non-contrast brain MRI showed CSF collection posterior to the third ventricle and congenital absence of the splenium of the corpus callosum, and polysomnography showed obstructive sleep apnea. The patient was treated with lisdexamfetamine and clonidine, showing improvement over time. Continuous positive airway pressure (CPAP) therapy led to marked improvement in sleep quality and daytime symptoms. This case highlights the potential mechanistic role of altered interhemispheric connectivity in ASD, a relatively underexplored area of the literature. Furthermore, it emphasizes the importance of individualized treatment as well as neuroimaging in atypical behavioral presentations. Further research is needed into the role of focal corpus callosum abnormalities in autism spectrum disorder (ASD).

## Introduction

The corpus callosum (CC) is the largest structure of white matter, connecting the two cerebral hemispheres. This structure, particularly the splenium, plays a central role in interhemispheric communication, and its disruption may contribute to these connectivity abnormalities [1,2]. Agenesis of the corpus callosum (AgCC) is a rare but well-recognized neurodevelopmental malformation. The epidemiology of this condition was first described by Myrianthopoulos in 1977 and is more frequently observed in individuals with developmental disorders. It is also noteworthy that the prevalence of isolated agenesis of the splenium of the corpus callosum remains uncertain, but corpus callosum malformations in some cases begin posteriorly (splenium and posterior body) [3]. Therefore, a proportion of partial agenesis is associated with the absence or hypoplasia of the splenium, and it may be caused by genetic and chromosomal anomalies, metabolic and toxic causes, intrauterine developmental disorders, hypoxic-ischemic insults, infections, and other causes [4,5].

The development of the corpus callosum typically begins at six weeks of gestation, when axons destined to cross the hemispheres do so medially. Then, at approximately twelve weeks, the first fibers cross at the level of the massa commissuralis (between the anterior and hippocampal commissures), finally forming the structure of the corpus callosum [6,7]. Most axonal tracts develop along non-neuronal cells of origin, such as glia, which guide the first pioneer axons toward their targets [7]. Several glial populations have been found to play an important role in the development of the corpus callosum. One such population, the so-called midline zipper glia, guides the midline fusion process, a necessary event in the period preceding corpus callosum formation. The term "glial wedge" refers to a population of glial cells that forms in the dorsomedial lateral ventricles, which, along with another glial population that forms in the region of the indusium griseum [8,9], plays an important role in the development of the corpus callosum. On the other hand, the "medial loop," composed mainly of migrating neurons, forms a bridge in the midline along which callosal axons reach the contralateral hemisphere. At twenty weeks, this part of the brain is completely formed. We must remember too that the corpus callosum is separated into parts, called rostrum, genu, body, isthmus, and splenium; and its functions are different, putting in the spotlight in this case that the splenium conjoins the temporal and occipital cortices on both sides [6]. A failure in this complex process explains many clinical presentations caused by agenesis or dysgenesis of this important structure.

The agenesis of the corpus callosum is a heterogeneous condition with a wide spectrum of clinical presentations. While some individuals present with significant neurological and developmental impairments, isolated forms of AgCC may be asymptomatic or associated with only mild deficits. Reported clinical features include intellectual disability (60%), visual impairment (33%), speech delay (29%), seizures (25%), and feeding difficulties (20%) [10]. In the matter of etiology, AgCC is a multifactorial condition. Genetic causes are identified in approximately 30-45% of cases, including chromosomal abnormalities (10%) and single-gene mutations (20-35%). In addition, environmental and metabolic risk factors, such as maternal alcohol exposure and maternal phenylketonuria, have also been implicated in its development [11].

In daily clinical practice, magnetic resonance imaging (MRI) has become the definitive tool for identifying and making sense of callosal anomalies. It is not just about confirming the absence of a structure; MRI allows us to map with precision whether we are dealing with complete agenesis or a more localized dysgenesis, each carrying its own clinical and genetic implications [12]. Beyond simple anatomy, neuroimaging is essential for distinguishing isolated findings from more complex syndromic presentations, such as Proud syndrome, where callosal malformation is a key piece of the neurological puzzle [11]. Especially in patients with atypical behavioral patterns, like those seen in autism spectrum disorder (ASD), the MRI provides a much-needed mechanical link between brain structure and clinical symptoms, ultimately guiding a more personalized therapeutic approach.

Studies have shown that up to one-third of individuals with AgCC present with autistic traits, particularly in domains related to social interaction, communication, and self-referential processing [8,13]. Neuroanatomical studies have revealed that individuals with ASD may exhibit partial agenesis or hypoplasia of the corpus callosum, especially in posterior regions such as the splenium, suggesting a shared underlying disruption of interhemispheric connectivity [14]. Postmortem analyses have identified reduced numbers of callosal axons, smaller axon diameters, and decreased overall cross-sectional area in individuals with ASD, which may reflect aberrant connectivity patterns relevant to both conditions [15]. Functionally, AgCC has been associated with impaired facial emotion recognition and deficits in theory of mind, mirroring core social cognition difficulties seen in ASD [16,17]. While not all individuals with AgCC meet full diagnostic criteria for ASD, the overlap in cognitive and behavioral features supports this possibility.

The presence of significant comorbidities, particularly attention deficit hyperactivity disorder (ADHD) and obstructive sleep apnea (OSA), may have further modulated the clinical phenotype. ADHD is commonly reported in individuals with agenesis of the corpus callosum, with approximately 19.3% having a formal diagnosis and up to 65.5% meeting at least one DSM-5 criterion [18].

The relationship between partial corpus callosum agenesis and autism spectrum disorder has been relatively underexplored, but there are some reports about this association. The presence of AgCC is considered a neurodevelopmental risk factor that increases a child's risk of developing autism [8,16]. Some studies reported that 18% to 33% of individuals with the condition have a formal diagnosis of autism spectrum disorder or exhibit related symptoms, and suggest that there is not only a shared behavioral similarity between AgCC and autism, but that there are shared genetic etiologies as well, but the association remains a topic of discussion.​​​​​​​

Another important point is that studies in the literature examining the relationship between ASD and ADHD, and between corpus callosum agenesis and disease prevalence, evaluate partial and complete corpus callosum agenesis together, without considering the isolated relationship in partial defects, such as the splenium. In the last year, some studies reported that decreased central corpus callosum volume in autistic children is associated with repetitive behaviors and motor skills [19], but the isolated part of the splenium is not clearly reported yet [20].​​​​​​​

## Case presentation

Case data collection

Data were obtained through a retrospective analysis of the patient's medical records from neurology and psychiatry clinic visits and interviews with the patient and his foster mother. Medical notes, developmental history, laboratory findings, physical and neurological examination results, and imaging reports from the records, covering the period from December 2017 to September 2025, were thoroughly reviewed. Written informed consent was obtained, and all data were fully anonymized. Ethical approval was secured in accordance with institutional standards for case report publication. This study was conducted in accordance with the CARE guidelines for case reports.

Analysis and current complaints

The patient was first brought to the neurology clinic by his stepmother in December 2017, at the age of thirteen. His primary complaints were threefold: irregular sleep disturbances, hypersomnolence, and progressively aggressive behavioral problems.

According to the foster mother, the patient was sleeping two to five hours per night. He had become increasingly disruptive, abusive, and combative. This course was reported to have significantly worsened after returning to a residential treatment facility in Richmond, VA, where he had stayed for ten months. The patient also reported visual hallucinations, such as "seeing green lights" and sensory disturbances.

Developmental and psychosocial history

The patient's early life history included significant challenges. When placed in foster care at two months old, he was found to be severely malnourished. His current foster mother became his foster mother when he was eight months old.

Developmentally, he began walking at fourteen months old, but due to frequent tripping and balance problems, he required physical therapy and dance-based interventions during childhood. His sensory development was quite atypical; pain sensation was reported that he did not consciously perceive pain until he was four years old.

From early childhood, the patient demonstrated persistent deficits in social communication and interaction. As a child, he was inconsolable with physical contact, showed reduced social reciprocity, and had difficulty engaging appropriately with others. Restricted and repetitive patterns of behavior were evident from early childhood, including constant rocking and head-banging behaviors in the crib. He was unable to lick food from a spoon until he received intensive occupational therapy, and despite sensory diet treatment, his food problems persisted. Socially, he was reported to have been bullied and highly suggestible. His foster mother stated that the patient "did not have an intellectual disability but needed time to learn at his own pace". Based on the developmental history and clinical features described above, the patient meets DSM-5 criteria for autism spectrum disorder.

Past medical and family history

The patient's current diagnoses included autism spectrum disorder (ASD) and ADHD (combined type). His surgical history included tonsillectomy. He had been taking lisdexamfetamine 30 mg for ADHD since the age of seven; his foster mother stated that this was the "only effective medication" for focus, but that the patient metabolized the medication rapidly. He was taking Clonidine 0.1 mg for sleep problems. His family history included a diagnosis of ADHD and a history of cocaine addiction in his biological mother.

No formal ASD or ADHD severity scales were available in the patient’s records; diagnoses were established clinically according to DSM-5 criteria. Functionally, the patient demonstrated significant behavioral dysregulation during childhood with subsequent improvement, and is currently able to maintain full-time employment with relative independence.

Physical and neurological examination

Clinical evaluation revealed the patient to be obese (BMI 27-28 kg/m²). Neurological examination revealed bilateral positive Marinescu's sign (palmomentonian reflex, characterized by the contraction of the ipsilateral mentalis muscle upon stimulation of the thenar eminence), poor eye contact, reduced verbal reciprocity and limited spontaneous conversational engagement, and mild difficulty with tandem gait.

Diagnostic evaluations

**Figure 1:**
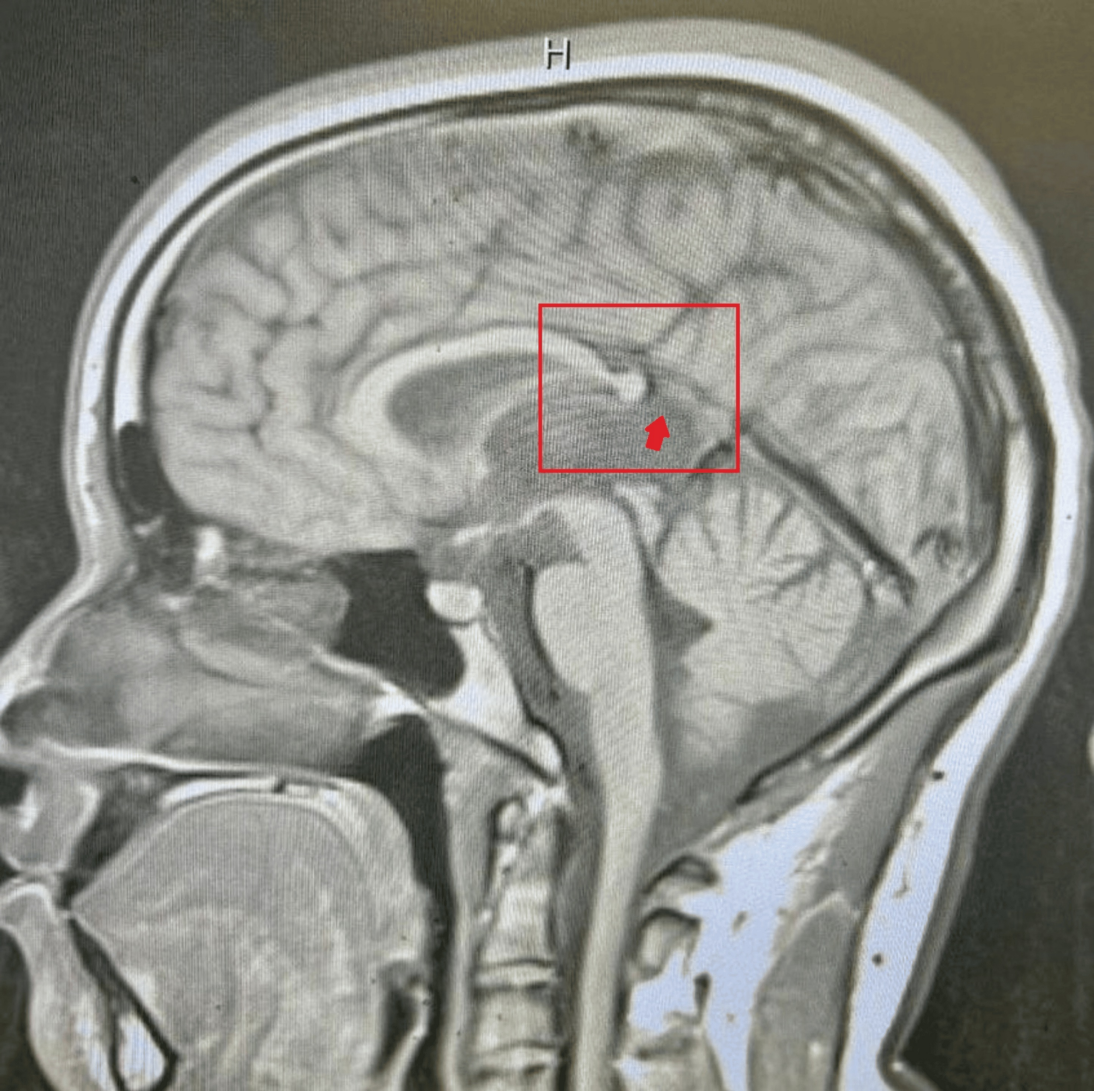
Sagittal T1-weighted MRI. Mid-sagittal T1-weighted MRI demonstrating truncation of the posterior corpus callosum. The splenium is absent (red rectangle), while the rostrum, genu, and body are preserved. The arrow indicates the expected location of the splenium, which is absent.

**Figure 2:**
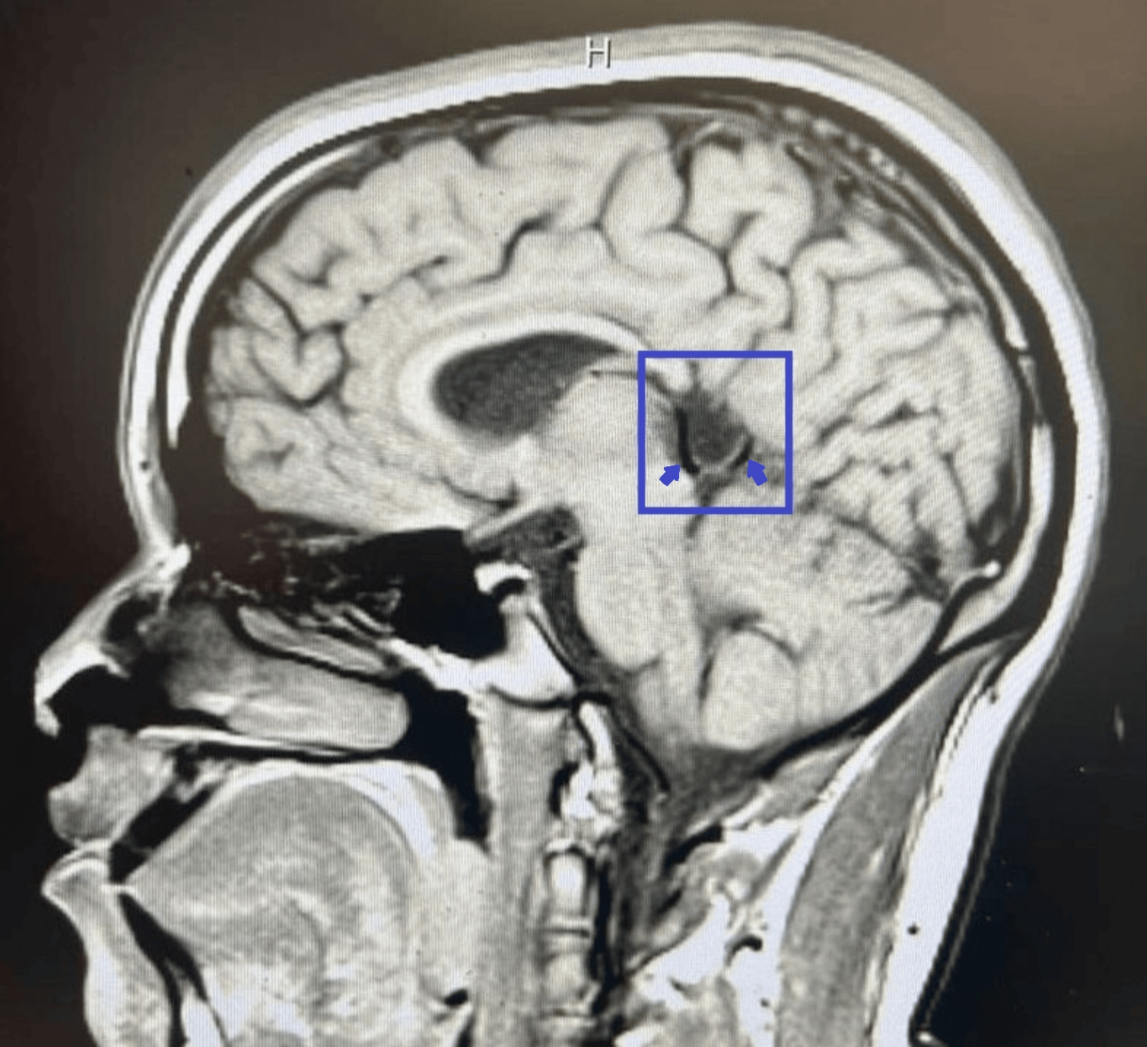
Sagittal T1-weighted MRI. Mid-sagittal T1-weighted MRI demonstrating absence of the splenium of the corpus callosum. A CSF-filled space is seen posterior to the third ventricle, communicating with it (blue rectangle). Arrows highlight the CSF-filled region.

**Figure 3:**
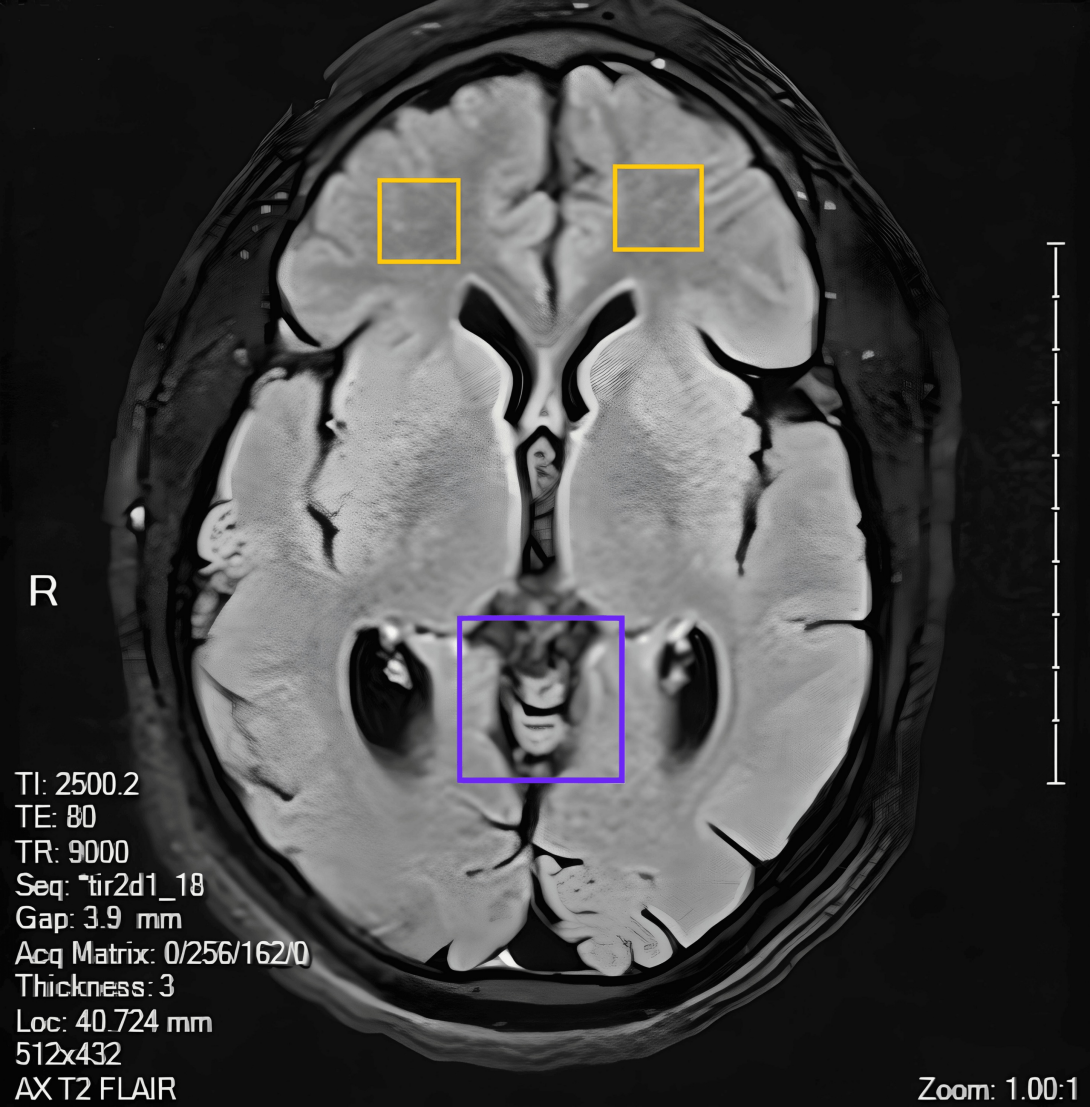
Axial T2/FLAIR MRI. Axial FLAIR MRI, the purple rectangle highlights a CSF-filled space in the posterior periventricular region, corresponding to the structure described posterior to the third ventricle on sagittal imaging. The yellow rectangles show subtle bilateral hyperintensities in the frontal white matter, consistent with nonspecific white matter changes, as described in the radiology report. FLAIR: fluid-attenuated inversion recovery.

**Figure 4:**
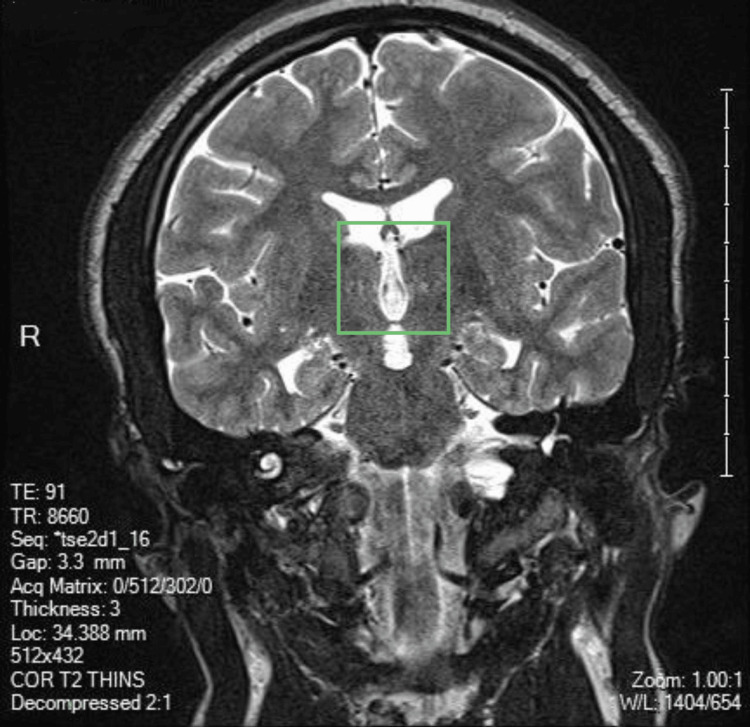
Coronal T2-weighted MRI. Coronal T2-weighted MRI. The third ventricle is indicated within the green rectangle. The image demonstrates symmetric hemispheric structures with no additional associated structural abnormalities at this level, complementing the findings of the posterior CSF-filled space.

Many studies were conducted on the patient. No magnetic resonance spectroscopy (MRS) or genetic testing was performed as part of the clinical evaluation. The diagnostic approach was based on clinical history, neurological examination, and structural neuroimaging, which were considered sufficient and determinant to characterize the presentation in this case and the underlying cause. The patient's MRI report, obtained on September 19, 2015, for "episodic mood disorder," revealed the main neuroanatomical finding: "Congenital absence of the splenium of the corpus callosum," no residual splenial tissue was identified, supporting true agenesis rather than hypoplasia, in which a rudimentary or thinned splenial structure would typically be present. The report also noted a "CSF collection approximately 2 cm wide behind the third ventricle" (unchanged compared with a 2010 CT scan and likely representing a congenital variant such as a cavum velum interpositum cyst) and "nonspecific diffuse T2/fluid-attenuated inversion recovery (FLAIR) signal abnormalities in the frontal deep white matter". No epileptiform activity was detected on the EEG requested for evaluation, but a polysomnography performed on December 25, 2017, confirmed the diagnosis of OSA, explaining the patient's complaints of hypersomnolence and snoring. A 2025 MRI reported bilateral nonspecific white matter changes in the frontal lobes, described as “ischemic changes” in the radiology report. These findings were not associated with focal neurological deficits on clinical examination and were considered nonspecific in the available reports. Representative brain MRI images in different slices and sequences are presented in Figures 1-4.

Table 1 displays a summary of the diagnostic tools and assessments that were used to clarify the patient's clinical profile and diagnosis.

**Table 1:** Diagnostic tools for assessment.

Diagnostic tool	Description
Developmental and Psychiatric Assessment	Diagnoses of autism spectrum disorder (ASD) and attention deficit hyperactivity disorder (ADHD) were made using DSM-5 criteria based on the patient's neurodevelopmental course, a detailed developmental history from the foster mother, and clinical observations.
Sleep Disorder Evaluation	Polysomnography (PSG) was performed on December 25, 2017, to objectively evaluate complaints of hypersomnolence and sleep disturbances. PSG confirmed the diagnosis of severe obstructive sleep apnea (OSA).
Neuroimaging	Brain magnetic resonance imaging (MRI) results were examined to evaluate central nervous system malformation. Imaging revealed the presence of isolated agenesis of the splenium of the corpus callosum.
Etiological Evaluation	A detailed history was taken to identify potential environmental risk factors associated with corpus callosum agenesis. This investigation revealed a history of cocaine use in the biological mother during pregnancy.
Pharmacological Monitoring	The effects and side effects of specific medications used for ADHD and sleep disorders (lisdexamfetamine and clonidine) were assessed through the patient's clinical follow-up notes.

Treatment and outcome

After the patient was diagnosed with OSA, continuous positive airway pressure (CPAP) treatment was initiated, and this resulted in a significant improvement in his sleep disturbances. Lisdexamfetamine and clonidine treatment was continued. With these combined treatments, the patient's behavioral problems improved over time.

At the time of the case report, the patient, 21 years old, was able to hold a full-time job and demonstrated a high level of functional autonomy despite severe difficulties in early childhood.

## Discussion

This case report documents a rare clinical presentation of isolated agenesis of the splenium of the corpus callosum (CC) in a 21-year-old, high-functioning male patient with autism spectrum disorder (ASD) and attention-deficit/hyperactivity disorder (ADHD). The absence of any identifiable splenial remnant on MRI further supports a diagnosis of true agenesis rather than hypoplasia. While the existing literature strongly supports the association between corpus callosum agenesis (AgCC) and ASD, most of these studies tend to combine cases of complete and partial agenesis. Our literature review also confirms the limited number of studies specifically focusing on the association between isolated agenesis of the splenium of the corpus callosum and ASD. This suggests that our findings help address a significant gap in the literature.

A potential etiological factor in this case is the reported history of prenatal cocaine exposure. However, this information is based solely on retrospective caregiver reports and cannot be independently verified; therefore, it should be interpreted with caution and cannot be considered a confirmed causal factor.

Disruption of long-range connectivity is a central hypothesis in the pathophysiology of ASD. While prenatal drug exposure has been associated with abnormalities in brain development, the specific evidence linking prenatal cocaine exposure to isolated splenial agenesis is limited. Therefore, any causal relationship in this context remains speculative [5]. This provides a plausible mechanistic framework linking the specific neuroanatomical finding in our case to the autistic characteristics exhibited by the patient.

The splenium, the posterior portion of the CC, plays a key role in the integration of interhemispheric visual, sensory, and socioemotional information [1], and its disruption may contribute to altered connectivity patterns observed in ASD. ASD is widely conceptualized as a disorder of altered brain connectivity, including reduced long-range and increased local connectivity. Recent advances in network neurosciences suggest that cognitive functions of higher order depend on the dynamic coordination of brain networks. This process is regulated by a large-scale control system; therefore, disruptions in these systems may contribute to ASD [20].

The presence of significant comorbidities, particularly ADHD and OSA, may have further modulated the clinical phenotype. Sleep fragmentation and chronic hypoxia associated with OSA have been implicated in executive dysfunction and behavioral dysregulation, which may further amplify neurodevelopmental vulnerabilities. CPAP treatment resulted in improved sleep quality, with a reduction in daytime sleepiness, improved alertness, and decreased irritability, highlighting the importance of screening for and effectively treating comorbidities in such complex neurodevelopmental cases. Treatment with lisdexamfetamine was associated with improved attention and reduced impulsivity, while clonidine contributed to better sleep initiation.

An additional clinically relevant feature in this case is the presence of visual perceptual disturbances, described as “seeing green lights.” Although these symptoms were transient and not persistent, they warrant consideration in the neuroanatomical context of splenial agenesis. The splenium plays a critical role in interhemispheric integration of visual information, and its absence may contribute to altered visual processing or perceptual disturbances. However, alternative explanations should also be considered, including nonspecific sensory phenomena, sleep-related disturbances, or neuropsychiatric manifestations associated with ASD. No epileptiform activity was identified on EEG, and the symptoms did not evolve into a defined psychotic or seizure disorder.

Additionally, the presence of bilateral frontal white matter changes on the 2025 MRI, described as “ischemic changes” in the radiology report, is an unexpected finding in a young adult and warrants cautious interpretation. In the absence of focal neurological deficits or a defined vascular event, these findings are more consistent with nonspecific white matter abnormalities rather than true ischemic injury. Potential contributing factors may include chronic sleep-disordered breathing with intermittent hypoxia, underlying neurodevelopmental vulnerability, or nonspecific imaging-related findings. However, no definitive etiology could be established in this case.

Taken together, these findings support a multifactorial model in which prenatal exposure, focal callosal abnormalities, and relevant comorbidities converge to shape a complex neurodevelopmental phenotype. These findings underscore the potential clinical relevance of isolated splenial defects in the neurobiology of ASD and raise the possibility that focal callosal abnormalities, rather than global agenesis, may be sufficient to alter brain network integration, particularly in systems underlying social cognition.

Despite these significant early neurodevelopmental challenges, the patient's high level of functional autonomy, achieved by the age of 21 while working full-time, demonstrates the success of individualized and multifaceted treatment. This case adds further support to existing evidence highlighting the relevance of focal callosal abnormalities, particularly isolated splenial defects, in the neurobiology of ASD.

## Conclusions

In conclusion, this case report provides supportive evidence of an association between isolated agenesis of the splenium of the corpus callosum and ASD, a relationship that remains underrepresented in the literature. Our findings support the notion that even focal callosal anomalies may contribute to the phenotypic spectrum of ASD. However, given the presence of multiple neurodevelopmental and environmental factors in this patient, including ADHD, obstructive sleep apnea, early malnutrition, and prenatal exposures, a direct causal relationship cannot be established.

These findings suggest that focal callosal abnormalities may contribute to the neurodevelopmental phenotype within a multifactorial context, rather than acting as an isolated determinant. Given the central role of the splenium in interhemispheric integration (particularly between temporal and occipital regions), further research is needed to better understand the specific impact of the isolated disruption of this structure. Notably, despite significant early neurodevelopmental challenges, the patient achieved a high level of functional autonomy, including maintaining full-time employment at 21 years of age. This highlights the variability of functional outcomes and underscores the importance of considering neuroimaging when evaluating atypical or treatment-resistant behavioral presentations. Further research is needed to better define how isolated agenesis of the splenium of the corpus callosum may contribute to complex neurodevelopmental disorders, even in high-functioning individuals. This case emphasizes the need for further research into focal corpus callosum abnormalities as a potentially underrecognized contributor to the neurobiological architecture of ASD, particularly in complex, multifactorial clinical presentations.
